# Spatial‒temporal heterogeneities of liver cancer and the discovery of the invasive zone

**DOI:** 10.1002/ctm2.70224

**Published:** 2025-02-09

**Authors:** Jiayan Yan, Zhifeng Jiang, Shiyu Zhang, Qichao Yu, Yijun Lu, Runze Miao, Zhaoyou Tang, Jia Fan, Liang Wu, Dan G. Duda, Jian Zhou, Xinrong Yang

**Affiliations:** ^1^ Department of Liver Surgery & Transplantation Liver Cancer Institute Zhongshan Hospital Fudan University Shanghai China; ^2^ Key Laboratory of Carcinogenesis and Cancer Invasion Ministry of Education Shanghai China; ^3^ Zhongshan‐BGI Precision Medical Center Zhongshan Hospital Fudan University Shanghai China; ^4^ College of Life Sciences University of Chinese Academy of Sciences Beijing China; ^5^ BGI‐Shenzhen Beishan Industrial Zone Shenzhen China; ^6^ Steele Laboratories for Tumor Biology Department of Radiation Oncology Massachusetts General Hospital and Harvard Medical School Boston Massachusetts USA

**Keywords:** immunosuppression, invasive zone, liver cancer, spatial heterogeneity, tumour microenvironment

## Abstract

Solid tumours are intricate and highly heterogeneous ecosystems, which grow in and invade normal organs. Their progression is mediated by cancer cells’ interaction with different cell types, such as immune cells, stromal cells and endothelial cells, and with the extracellular matrix. Owing to its high incidence, aggressive growth and resistance to local and systemic treatments, liver cancer has particularly high mortality rates worldwide. In recent decades, spatial heterogeneity has garnered significant attention as an unfavourable biological characteristic of the tumour microenvironment, prompting extensive research into its role in liver tumour development. Advances in spatial omics have facilitated the detailed spatial analysis of cell types, states and cell‒cell interactions, allowing a thorough understanding of the spatial and temporal heterogeneities of tumour microenvironment and informing the development of novel therapeutic approaches. This review illustrates the latest discovery of the invasive zone, and systematically introduced specific macroscopic spatial heterogeneities, pathological spatial heterogeneities and tumour microenvironment heterogeneities of liver cancer.

## Graphical abstract

Solid tumours are complex ecosystems characterised by significant heterogeneity in both their component composition and spatial‒temporal organisation. This complexity contributes to challenges in accurate stratification, as well as in the development of drug resistance and early relapse. Our study maps the transcriptional architecture of human liver cancer using high‐resolution Stereo‐seq and single‐cell RNA sequencing across various tissue types, and we identified a 500 µm‐wide zone centred around the tumour border in patients with liver cancer, referred to as ‘invasive zone’. We detected strong immunosuppression, metabolic reprogramming and severely damaged hepatocytes in this zone. Similarly, the invasive zone between tumour tissues and paratumoural tissues of solid tumours resembled the border area of the two opposing powers, namely, ‘Chu’ and ‘Han’ Dynasties in fighting for hegemony in the late Qin Dynasty. The most intense heavy fighting and complex mutual interactions were found around the battlefronts of the two powers, which was called ‘Honggou’, namely, the wide chasm in the history like ‘invasive zone’. Furthermore, when the smoke of war gradually dissipated in the long river of history, the border area between Chu and Han Dynasties was forever fixed on the Chinese chess board and represented the balance between offense and defense.

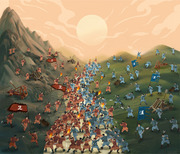

## 1 INTRODUCTION

Primary liver cancer, a deadly liver disease, significantly affects the survival and quality of life of millions of patients globally. According to statistics from the National Cancer Center of China, approximately 367 700 new cases of liver cancer are diagnosed annually, accounting for 40% of cases worldwide; liver cancer is the fifth most frequent malignancy in China.^1^, ^2^, ^3^, ^4^ Simultaneously, liver cancer stood out as the second most common cause of cancer‐related death in China (316 500 deaths in 2022), representing 40% of all cancer‐related deaths worldwide.^1^, ^2^, ^3^, ^5^ The 5‐year survival rate of patients with liver cancer in China is only 14.1%. Pathologically, liver cancer is classified into three primary subtypes: hepatocellular carcinoma (HCC), intrahepatic cholangiocarcinoma (ICC) and combined HCC‒ICC. These subtypes exhibit distinct epidemiology, clinicopathological features, oncogenic drivers and clinical management approaches.^6^, ^7^, ^8^, ^9^ All liver cancers progress rapidly with high rates of distant metastasis, and tumour recurrence after treatment is frequent. These unfavourable features are due to their complex and heterogeneous ecosystems, where the cancer cells interact closely with diverse cell types and the extracellular matrix.^10^, ^11^ Accumulating studies revealed spatial heterogeneities of liver cancer especially in margin areas and tertiary lymphoid structures (TLS).^12^, ^13^ Tumour immune barrier structure formation of SPP1^+^ macrophages and cancer‐associated fibroblasts (CAFs) limits the efficacy of immune therapy in HCC and dominant TLS in paratumour tissue instead of in tumour tissue featuring with immature phenotype represented immune excluded states with worst prognosis of ICC patients.^14^, ^15^ To comprehensively understand the mechanisms of tumour progression and metastasis and develop more effective treatments, it is crucial to dissect the cancer ecosystem across spatial and temporal dimensions. This includes investigating tumour margin areas where tumour cell infiltration and invasion occur. The heterogeneity of liver cancer can be categorised into four aspects: inter‐tumour, intra‐tumour, temporal and spatial heterogeneity. Advances in high‐throughput sequencing technology have significantly contributed to the fine dissection of spatial‒temporal heterogeneity in liver cancer, particularly for spatial heterogeneity.

## 2 DISCOVERY OF INVASIVE ZONE AND ITS CLINICAL IMPLICATIONS

The margin areas around the boundary between tumour tissue and peritumoural tissue, where tumour cells invade peritumoural tissue and establish direct contact, are crucial regions in solid tumours for understanding tumour progression and metastasis.^10^, ^16^ First, the proposed guidelines by the International Immunooncology Biomarkers Working Group for evaluating tumour‐infiltrating lymphocytes in solid tumours defined the ‘invasive margin’ as a 1‐mm region centred on the boundary between malignant cell nests and surrounding tissue.^17^ Nevertheless, this definition lacks evidence illustrating the heterogeneity of invasive margins. Therefore, gaining a comprehensive understanding of the components, their spatial heterogeneities, and the interactions between tumour cells and the tumour microenvironment (TME) in this area will enhance insights into tumour invasion and metastasis, prognosis, and developing novel therapeutic strategies for solid tumours.

**FIGURE 1:**
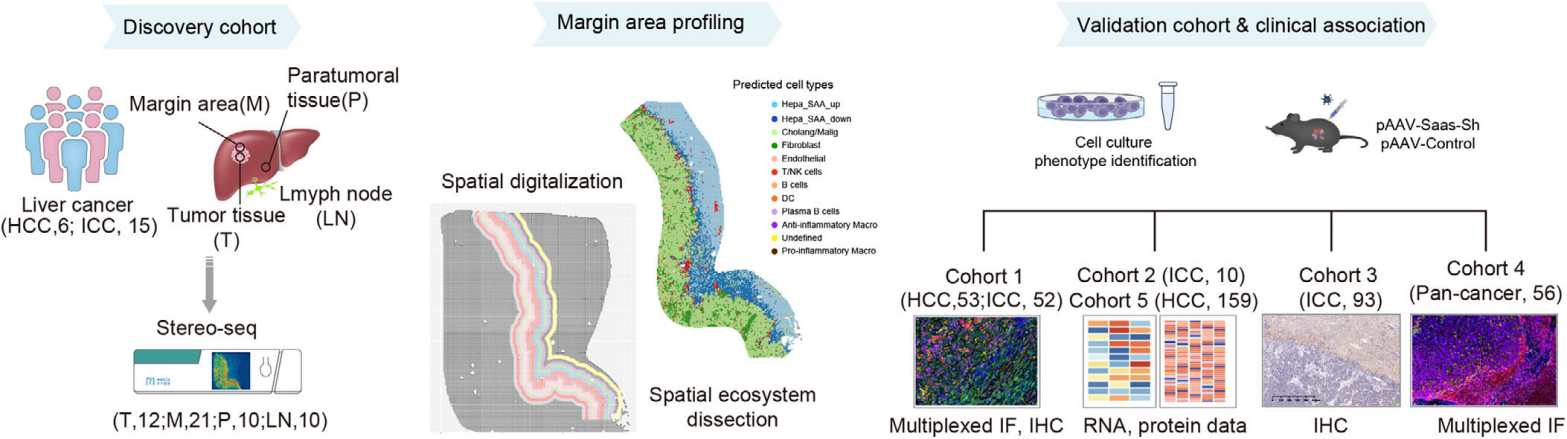
Schematic diagram of the workflow of spatial transcriptomics acquisition of liver cancer, margin area profiling and further data analysis.

Our study^12^ characterises the transcriptional architecture of human liver cancer using high‐resolution Stereo‐seq and single‐cell RNA sequencing (scRNA‐seq) across various tissue types, including tumour, margin area paratumour and lymph node (LN) samples. The analysis involved 98 Stereo‐seq datasets from 53 samples obtained from 6 HCC patients and 15 ICC patients (Figure 1). A novel segmentation method, namely SDM, was used to dissect the local microenvironment at the tumour margins, dividing the area into layers for detailed cell component analysis. In vivo experiments using mouse models of HCC and colon adenocarcinoma liver metastasis demonstrated that Adeno‐Associated Virus (AAV)‐mediated knockdown of Saas in hepatocytes reduced macrophage accumulation around the tumour border and slowed tumour growth. The findings were further validated across five cohorts, including primary liver cancer and metastatic cases (423 patients).

**FIGURE 2:**
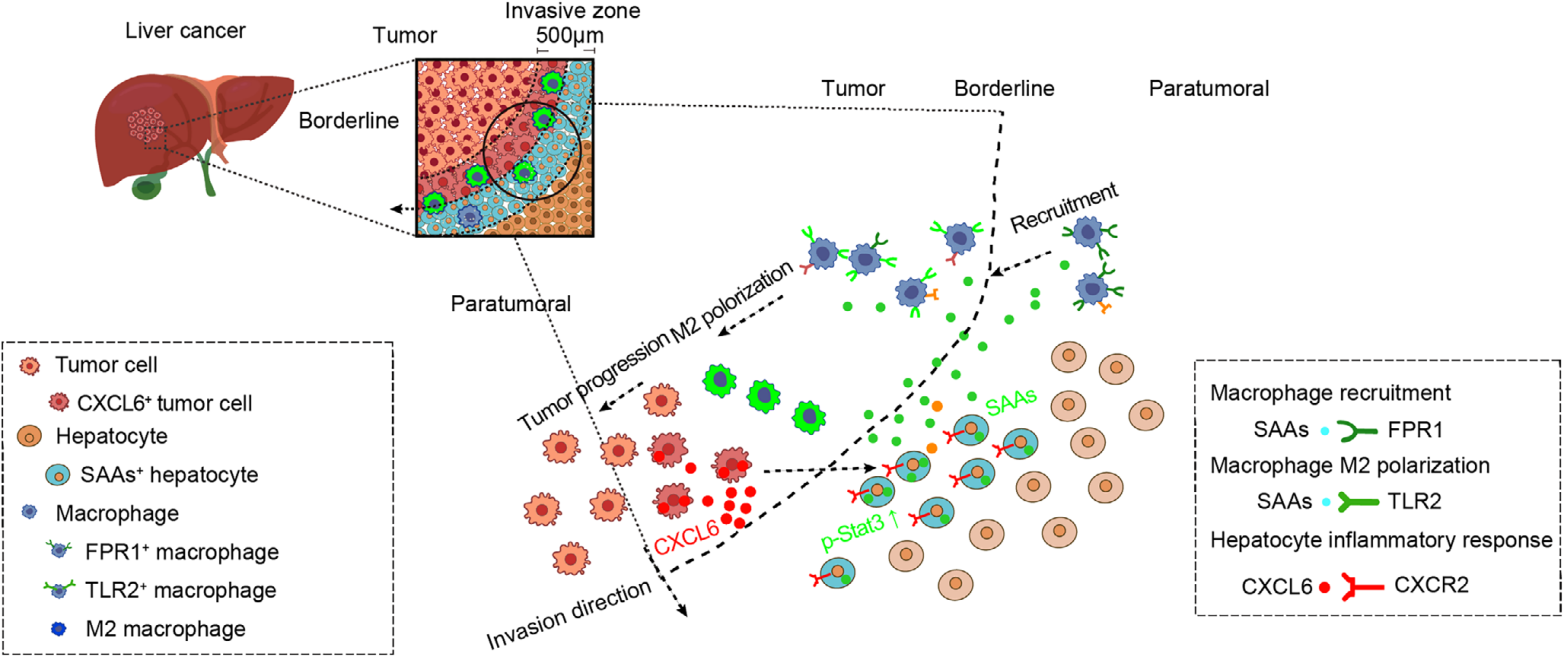
Local ecosystem of the invasive zone in liver cancers.

Our study using a novel tumour border scanning and digitisation model, facilitated by nanoscale resolution‐SpaTial Enhanced REsolution Omics‐sequencing (Stereo‐seq) developed by BGI, revealed a 500 µm‐wide zone centred around the tumour boundary in human liver cancers, which is termed the ‘invasive zone’^18^, ^19^ (Figure 2). Strong immunosuppression, metabolic reprogramming and severely damaged hepatocytes were detected within this zone. Subpopulations of damaged hepatocytes exhibiting increased expression of serum amyloid A1 and A2 (SAA1 and SAA2, collectively referred to as SAAs) were identified near the tumour border on the peritumour side. CXCL6 overexpression in adjacent malignant cells induces JAK‒STAT3 pathway activation in hepatocytes, which subsequently causes SAA overexpression. Moreover, the overexpression and secretion of SAAs by hepatocytes within the invasive zone can recruit tumour‐associated macrophages (TAMs) and induce their M2 polarisation, thereby fostering local immunosuppression in the invasive zone, likely promoting to tumour progression. In vivo experiments using tumour mouse models confirmed that the knockdown of SAAs in hepatocytes reduced macrophage accumulation around the borderline and delayed tumour growth. These findings were further confirmed in four additional independent cohorts of patients with primary and secondary liver cancer. Recently, He et al.^20^ discovered that SAAs prompted neutrophils to express programmed cell death ligand 1 (PD‐L1) by activating the glycolytic pathway through the LDHA/STAT3 pathway. This process also led to the release of oncostatin M, which in turn diminished the function of cytotoxic T cells. Due to the invasion of tumour cells in the invasive zone, hepatocytes are destroyed, triggering an acute phase response marked by elevated expression of SAAs. This, in turn, leads to macrophage recruitment and M2 polarisation. In clinical cohorts, the expression levels of SAAs and the extent of SAA‐positive zones can be used to categorise the invasive zone into either active or silent type with different prognosis. In mouse models, knockdown of Saas also inhibited the growth of HCC tumours and the metastasis of colorectal cancer to the liver, highlighting potential therapeutic targets for clinical practice.

Actually, 500 µm‐wide zone centred on the tumour border was distinct area different from other regions in tumour tissue or paratumoural tissue and partial hepatectomy for liver cancers with a resection margin aiming grossly at 1–2 cm was sufficient in preoperative 3D imaging and in surgical procedures.^21^ In clinical practice, the invasive zone can be identified using the immunohistochemical staining of SAAs in margin areas, which reveals the inflammatory response, macrophages infiltration and tumour progression of liver cancers.

To the best of our knowledge, our study was the first to map the transcriptional architecture and characterise the spatial heterogeneity of the TME using nanoscale spatial transcriptomics. The concept of an ‘invasive zone’ was defined based on the unique characteristics of tumour cells, stromal cells and the immune microenvironment in the 500 µm‐wide zone centred on the borderline. These findings underscore the significant promise of high‐resolution spatially resolved transcriptomics to provide valuable biological insights for developing novel therapeutic approaches against solid tumours.

A subsequent study provided a more detailed analysis of the immunosuppressive microenvironment within the invasive zone. Ruf et al.^22^ observed low infiltration of mucosal‐associated invariant T cells (MAIT) in tumour tissues and high infiltration of MAIT around the boundary of the tumour tissue. Further investigation indicated that CD163^+^ PD‐L1^+^ macrophages primarily located in adjacent liver tissue impaired MAIT cell function through direct contact and PD‐L1‐dependent mechanisms, leading to reduced interferon‐gamma (IFN‐γ) production at the tumour‐to‐liver interface.

## 3 MACROSCOPIC FEATURES OF SPATIAL HETEROGENEITY IN LIVER CANCER

**FIGURE 3:**
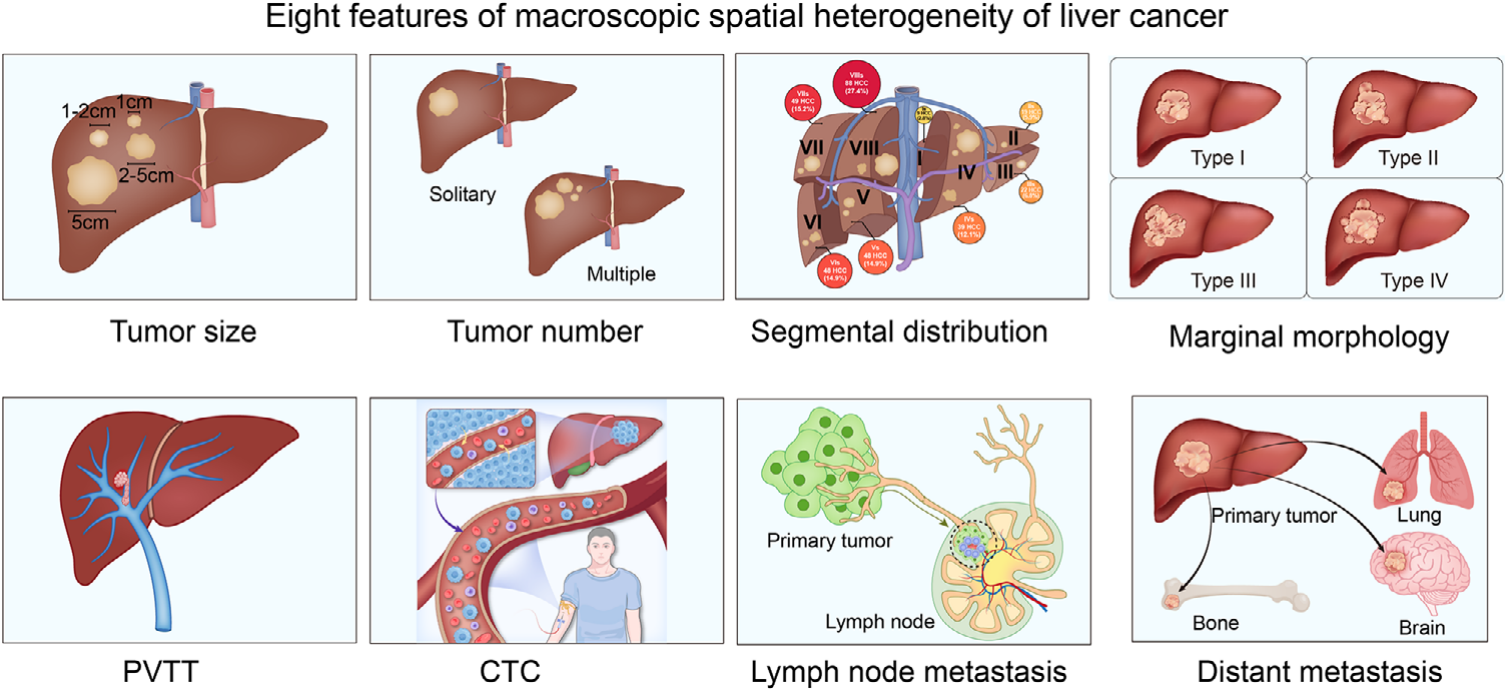
Macroscopic features of spatial heterogeneity in liver cancer.

Liver cancers exhibit notable spatial heterogeneity in their macroscopic morphology. Spatial heterogeneities include variations in tumour pattern (tumour size, tumour number and segmental distribution), vascular invasion, marginal morphology, portal vein tumour thrombosis (PVTT), circulating tumour cells (CTCs), LN metastasis and distant metastasis. These variations play a critical role in establishing an accurate diagnosis and prognosis of the disease and designing optimal treatment strategies (Figure 3). Genetic mutations in the neoplastic subclones in the liver enable them to compete within the basic structure of the hepatic lobule, establishing specialised niches that form tumour ecosystems. In liver cancer, cancer cells actively create ecological niches to gain fitness advantages. This involves manipulating the surrounding liver environment, such as creating an immune suppressive environment for immune evasion, co‐opting fibroblasts to provide growth factors and secrete matrix components, and stimulating angiogenesis to create a vasculature that provides oxygen and nutrients.^23^

To classify HCC, the most widely used is the Barcelona Clinic Liver Cancer (BCLC) system, which primarily considers liver function, tumour size, tumour number, vascular invasion and distant metastasis.^24^ HCCs less than 1.0 cm and 1.0–2.0 cm in diameter are categorised as subcentimetre HCC (scHCC) and small HCC, respectively. Patients with scHCC exhibit an impressive 5‐year survival rate of up to 98.5%, which significantly surpasses those with HCCs with a diameter of 1.0–2.0 cm (89.5%).^25^ Tumours smaller than 1 cm were associated with lower overall liver cancer recurrence than larger tumours. Conversely, tumour sizes larger than 5 cm, the presence of multiple tumours, and satellite lesions around solitary tumours indicate higher HCC malignancy and worse prognosis.^26^

Although no significant histological differences were observed between the left and right liver lobes, they exhibited distinct transcriptomic characteristics. Proteome analysis indicated that upregulated proteins expressed in the left lobe were mainly associated with energy metabolism processes, such as oxidative phosphorylation and the Tricarboxylic Acid Cycle (TCA)Alpha‐Fetoprotein cycle, whereas small‐molecule metabolism processes were substantially enriched in the right lobe.^27^ Using the standard reference atlas, patients with HCC were categorised into three subtypes based on segmental criteria. The C1 subtype exhibited strong enrichment of ribosome biogenesis and was predominantly located in the right lobe. The C2 subtype displayed an intermediate phenotype, whereas the C3 subtype was associated with neutrophil degranulation and was mainly found in the left lobe.^27^ In addition, the segmental distribution of liver tumours in the liver was associated with microvascular invasion (MVI), tumour recurrence and patient survival, with segment eight most commonly involved and segment one least frequently involved.^28^, ^29^

Numerous studies have identified heterogeneities in the marginal morphology of liver cancer and their associations with genomic profiles and patient prognosis. As early as 1987, Moriyama's group classified HCCs into five gross subtypes based on macroscopic shape: single nodular type, single nodular with extranodular growth, contiguous multinodular, infiltrative and early HCC.^30^, ^31^ Fan et al.^32^ identified distinct features of gross marginal morphology among patients with solitary HCC, including margin morphology classification (MMC)‐I (smooth type), MMC‐II (extranodular growth type) and MMC‐III (infiltrative type) with unique transcriptional modules. In particular, survival analysis revealed that infiltrative‐type HCC exhibited higher vascular invasion, microsatellite proportion and TP53 mutation, indicating a better response to adjuvant transarterial chemoembolization (TACE) than other HCC types.

PVTT is associated with aggressive tumour biology, characterised by high tumour grade, significant tumour burden (both in number and size of lesions), elevated serum tumour markers such as alpha‐fetoprotein (AFP), impaired liver function (as indicated by abnormal liver function tests) and poor patient performance status. The BCLC staging system classifies HCC patients with PVTT as being in advanced stage (BCLC stage C), reflecting the severe prognosis and challenging treatment landscape for these patients^33^, ^34^, ^35^, ^36^ (Figure 3).

CTCs are cancer cells that have shed from the primary tumour into the bloodstream, providing a non‐invasive biomarker for monitoring liver cancer. Recent advances in CTC detection and characterisation have enhanced the understanding of their role in liver cancer metastasis and progression. By comparing CTCs from adjacent vascular locations using scRNA‐seq, spatiotemporal transcriptional dynamics related to cell cycle regulation and immune evasion during their transport through the bloodstream could be traced.^37^ Notably, CCL5 was found to play a crucial role in CTC immune evasion by recruiting Tregs through the transforming growth factor (TGF)‐β1‐p38‐MAX signalling pathway. Clinically, CTCs serve as a prognostic marker for poor survival and recurrence in liver cancer patients, guiding treatment decisions and enabling personalised therapeutic strategies (Figure 3).

LN metastasis plays a crucial role in AJCC tumour staging in liver cancer and serves as a significant basis for predicting unfavourable outcomes. Its persistence also contributes significantly to further distant metastasis and, ultimately, to patient death.^7^, ^38^ HCC infrequently metastasizes to the LNs, whereas ICC metastasizes to the LNs in approximately one‐third of all cases.^39^ During LN metastasis, tumour cells evolve into a cell population with greater epithelial‒mesenchymal transition (EMT) features, higher heterogeneity and immune evasion.^40^, ^41^ Metabolic adaptations in tumour cells during LN metastasis were also observed. Lee et al.^42^ conducted comparative transcriptomics and metabolomics analyses using mouse models and revealed that LN metastasis requires tumour cells to undergo a metabolic shift towards fatty acid oxidation. Furthermore, tumour cells located near the invasive front of LN metastasis exhibited selective transcriptional activation of the coactivator Yes‐associated protein (YAP), leading to the upregulation of genes involved in the fatty acid oxidation signalling pathway. The authors generated multi‐omic profiling of 257 primary and 176 metastatic regions from 182 patients with HCC and found that primary tumours rich in hypoxia signatures facilitated polyclonal dissemination.^43^ In addition, subclones lacking Wnt mutations demonstrated greater selectivity for metastasis than those harbouring Wnt mutations, and they were associated with an activated fibroblast‐rich microenvironment. Immunosuppressive B cells enriched in metastases without Wnt mutations compromise antitumour immunity by inducing terminal exhaustion of CD8^+^ T cells.

## 4 PATHOLOGICAL FEATURES OF SPATIAL HETEROGENEITY IN LIVER CANCERS

**FIGURE 4:**
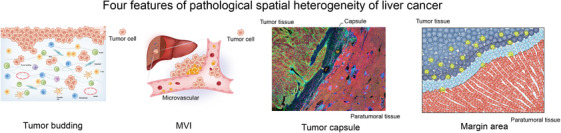
Pathological features of spatial heterogeneity in liver cancer.

Four significant pathological features highlight spatial heterogeneity in liver cancers: tumour budding, MVI, tumour capsule and margin area (Figure 4).

Tumour budding, defined as the presence of isolated single or up to four cancer cell clusters at the invasive margins of the tumours, has emerged as a practical and significant histological index for identifying lesions with higher malignant potential across various tumour types.^44^, ^45^ Increasing evidence indicates that budding cancer cells around the invasive margin exhibit elevated expression of EMT‐related transcription factors, such as ZEB1/2, TWIST1/2 and SNAI1/2, along with reduced cell adhesion and decreased E‐cadherin expression in liver cancers, both in HCC and ICC.^46^, ^47^ Moreover, the increased number of tumour budding in HCC was significantly correlated with high T‐cell counts, macrophage infiltration and programmed cell death protein 1 (PD1) expression.^48^ Clinically, a higher degree of tumour budding correlates with more aggressive tumour behavior, including increased metastasis, recurrence and poor overall survival (OS).^49^ Furthermore, the number of tumour budding cells is associated with an immunosuppressive microenvironment, characterised by increased T‐cell infiltration, macrophage accumulation and upregulated PD1 expression, which further contributes to tumour progression and immune evasion. This highlights tumour budding as a potential biomarker for prognosis and a target for therapies aimed at modulating the tumour‒immune interface.

In addition, MVI, defined as nests of malignant cells lining the vascular cavities of endothelial cells or the portal and hepatic venous systems, was frequently observed in minor portal vein branches within the tumour capsule and peritumoural liver tissue in microscopic view for 15%–57% of all HCC patients.^50^, ^51^ Li et al.^52^ conducted a scRNA‐seq analysis of HCCs and discovered that MVI^+^ malignant cells exhibited upregulation of genes enriched in pathways such as E2F targets, MYC targets v1, protein secretion, the G2M checkpoint and DNA repair. Clinically, the presence of MVI is a strong predictor of early recurrence and reduced survival, as it reflects the propensity of tumour cells to invade the vasculature, disseminate and establish metastatic lesions.^53^, ^54^ It is also a key factor in determining the suitability of liver transplantation and resection strategies, as MVI‐positive patients often require more intensive surveillance and adjuvant therapies.

Macroscopic and pathological investigations have shown that the tumour capsule are present around tumours in 10%–76% of patients with HCC, but are rarely observed in patients with ICC.^55^, ^56^ These fibrous capsules result from interactions between the tumour and host tissues and serve as physical barriers that prevent immune cell infiltration into the margin area, thereby hindering the interplay among the cells around the border.^56^, ^57^ However, some patients with HCC and almost all patients with ICC lack a tumour capsule around the boundary of tumour tissue.

Numerous studies have demonstrated that the margin area, recognised as the most active part of liver cancer, is enriched with immune cells such as CCR1^+^ CD14^+^ monocytes, CD20^+^ B cells, CD4/CD8 double‐positive T cells, etc.^48^, ^58^ In addition, the condition of the margin area influences tumour progression and recurrence in patients with liver cancer.

## 5 HETEROGENEITY OF IMMUNE TME IN LIVER CANCERS

The TME in liver cancers is characterised by a complex interplay between antitumour and protumour factors.^59^ Within this environment, diverse immune cells interact with tumour cells, significantly influencing tumour progression. Infiltrating lymphocytes such as CD8^+^ and CD4^+^ T cells, Tregs, TAMs, tumour‐associated neutrophils (TANs), myeloid‐derived suppressor cells (MDSCs) and natural killer (NK) cells can exhibit antitumour or pro‐tumour effects, depending on the context, thereby influencing tumour progression. scRNA‐seq technology has revealed distinct functional subtypes of T cells in HCC, including exhausted CD8^+^ T cells and Tregs preferentially enriched and clonally expanded in tumour tissues and innate‐like CD8^+^ T cells, namely, CD161^+^ CD8^+^ T cells, with low cytotoxicity and clonal expansion, leading to compromised antitumour immunity and early relapse.^6^, ^60^, ^61^ Furthermore, single‐cell technologies have revealed that TANs have a dynamic spectrum of pro‐tumour and antitumour functions. Xue et al.^62^ demonstrated through in vitro induction of TANs and ex vivo analyses of patient TANs that CCL4^+^ TANs can recruit TAMs, whereas PD‐L1^+^ TANs can suppress T‐cell cytotoxicity. In pan‐cancer immune research, HLA‐DR^+^CD74^+^ neutrophils have been identified as alternative antigen‐presenting cells (APCs) in diverse cancer types and can present tumour neoantigens to T cells, thereby reshaping the T‐cell immune pool and enhancing antigen‐specific responses.^63^ Increasing evidence indicates that tumour‐infiltrating B cells and plasma cells, collectively known as tumour‐infiltrating B lymphocytes (TIL‐Bs), enhance antitumour immunity by uniquely presenting antigens to T cells. They also play a crucial role in assembling and perpetuating an ‘immunologically hot’ TME.^64^, ^65^ Ma et al.^66^ revealed the significant heterogeneity within B and plasma cells and identified two independent developmental pathways to antibody‐secreting cells: the canonical germinal centre (GC) and the alternative extra‐follicular (ET) pathway. They observed an obvious cancer‐type preference, with colon adenocarcinoma and HCC representing cancers enriched for the GC and EF pathways, respectively.

**FIGURE 5:**
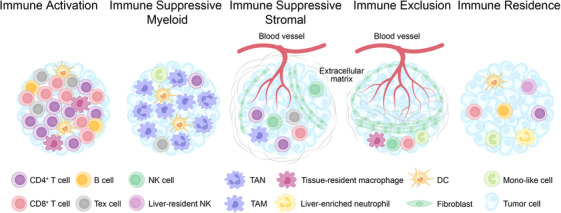
Spatial heterogeneity of immune cell components in tumour microenvironment (TME) of liver cancer.

Extensive scRNA‐seq analyses of liver cancer samples (including HCC, ICC and combined HCC‒ICC) classified the immune microenvironment into five subtypes: immune activation, myeloid, immune suppressive stromal, immune exclusion and immune residence. Each subtype exhibits distinct cellular communication networks, tumour cell mutations and transcriptomic information^62^ (Figure 5). Notably, the myeloid‐enriched immunosuppressive and matrix‐enriched immunosuppressive subtypes were associated with improved prognosis, whereas the immunoresident subtype was associated with worse prognosis.

**FIGURE 6:**
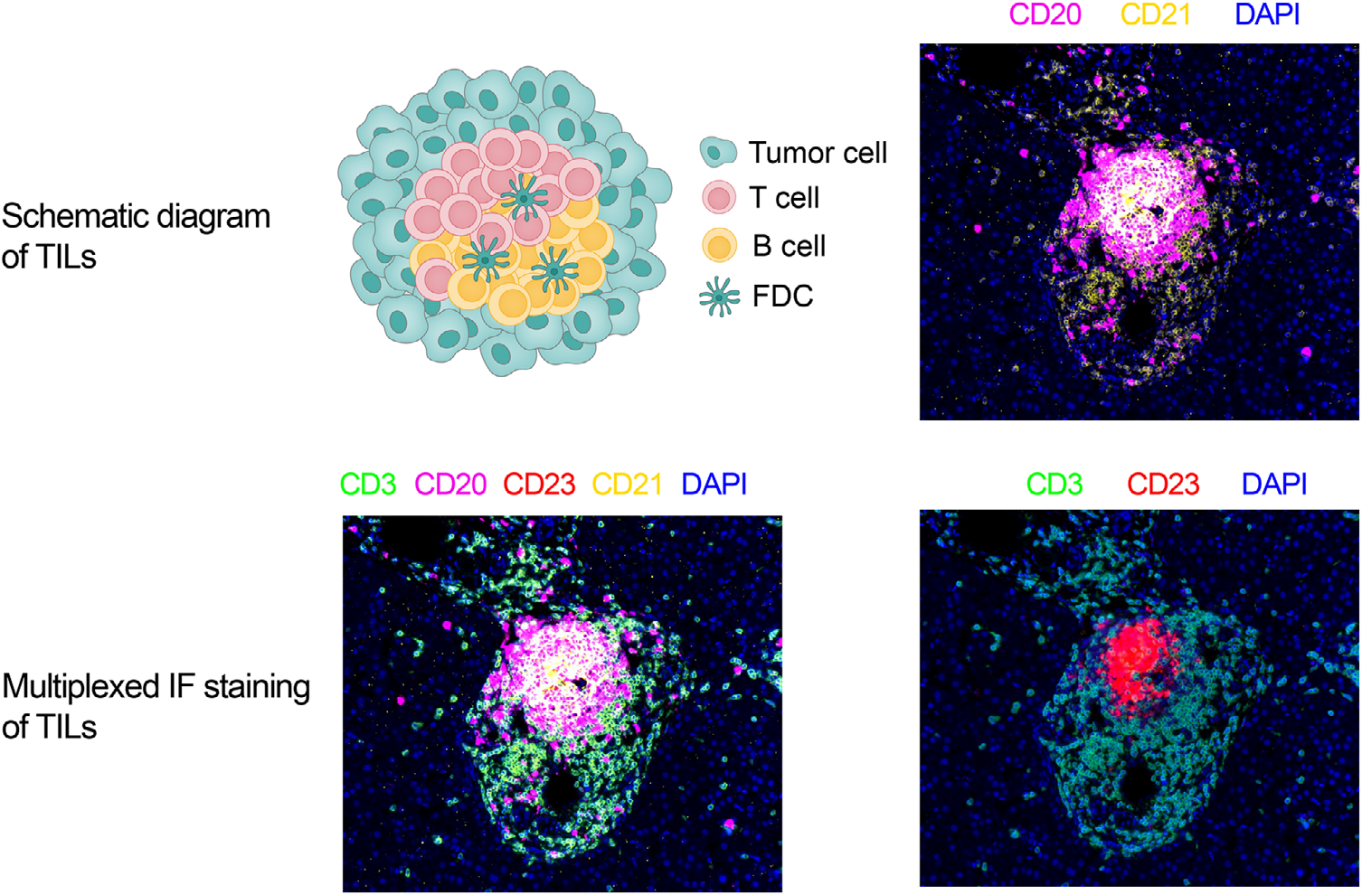
Cellular components and tumour‐infiltrating lymphocyte (TIL)‐multiplexed Immunofluorescence (IF) staining.

Spatial heterogeneities within the TME lie in the spatial distribution of immune cells across hot/cold tumour phenotypes, as well as around invasive margins and TLS. For instance, in human colorectal tumours, the density of T cells (CD3, CD8, GZMB and CD45RO expression) within the tumour centre and invasive margins indicates a strong or weak adaptive immune response in situ, which is associated with tumour recurrence and patient survival.^67^ Moreover, the level and spatial distribution of CD3^+^ CD8^+^ T‐cell infiltration categorises solid tumours into four distinct phenotypes: hot (inflamed), altered (divided into excluded or immunosuppressed) and cold (non‐inflamed).^68^, ^69^ ‘Immunologically hot’ tumours, characterised by a high degree of T‐cell infiltration, present a more favourable environment for immune checkpoint inhibitors (ICIs), such as anti‐PD1 and anti‐CTLA4, alone or combined with other therapies.^69^ Zheng et al.^70^ employed time‐of‐flight mass cytometry (CyTOF) to analyse the immune microenvironment of HCC across different areas. They observed that CD4/CD8 double‐positive T cells were notably enriched at the invasive margin and exhibited higher levels of IFN‐γ, tumour necrosis factor‐alpha (TNF‐α) and PD‐1 upon stimulation. Pathological examinations of HCC indicated that infiltrating CD20^+^ B cells were predominantly located in the tumour invasive margin, and they were characterised by a typical memory phenotype compared with intra‐tumour and peritumour areas.^58^ In addition, a high density of margin‐infiltrating B lymphocytes was positively correlated with less malignancy, heightened CD8^+^ T‐cell infiltration and improved prognosis. Liu et al.^71^ discovered that CCL15, the most abundant chemokine in human HCC, recruited CCR1^+^CD14^+^ monocytes to the HCC invasive margin. These monocytes exhibited higher expression of PD‐L1, B7‐H3, T‐cell immunoglobulin domain and mucin domain‐3, contributing to immunosuppression (Figure 6).

**FIGURE 7:**
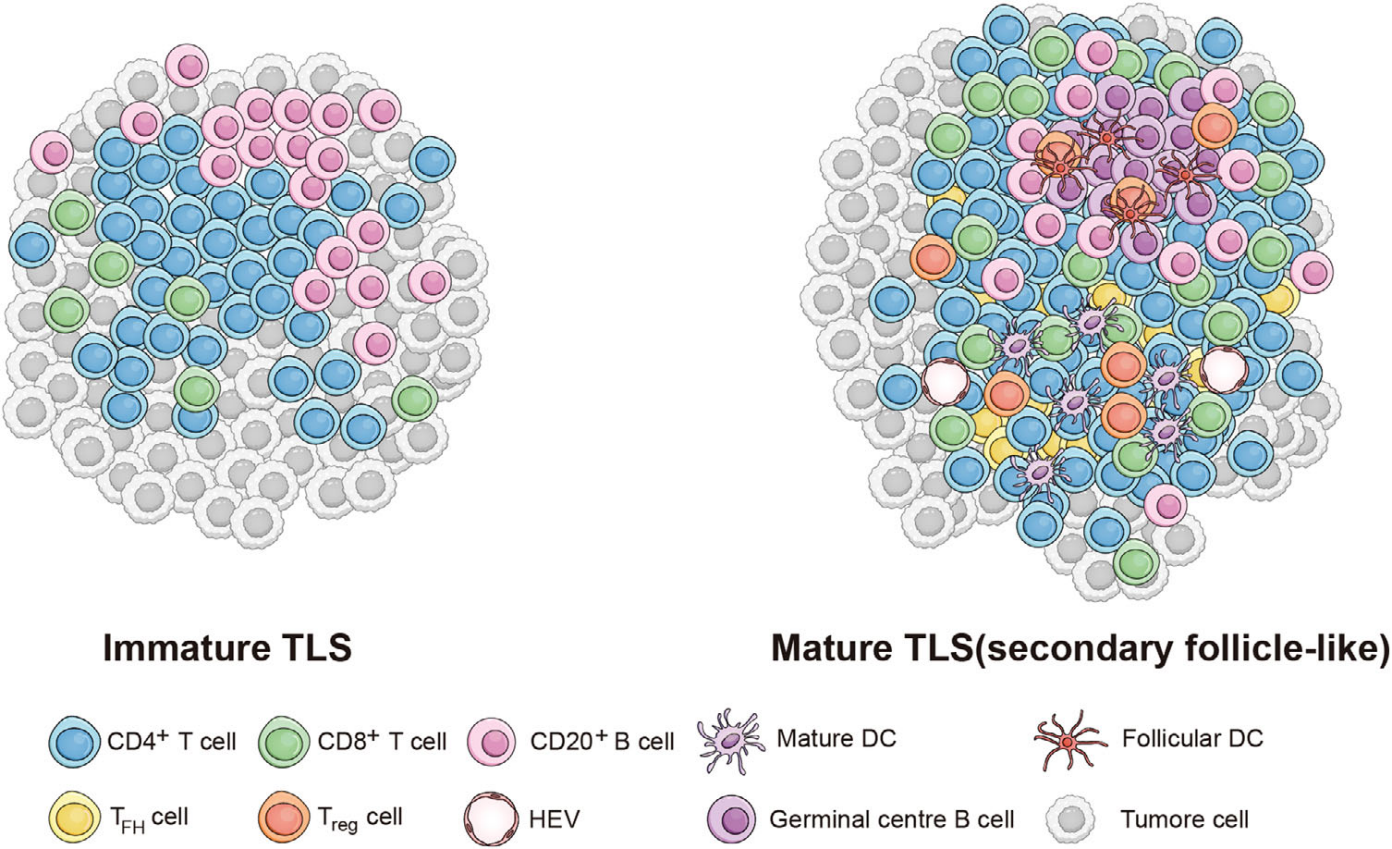
Maturity of tumour‐infiltrating lymphocyte (TIL) and their cellular components.

Ding et al.^72^ conducted a preliminary investigation into the distribution of TLS in ICCs. They categorised TLS into four types based on their composition and spatial distribution, revealing that a higher presence of TLS within the tumour correlated with improved patient prognosis, whereas increased TLS in peritumoural tissues correlated with worse prognosis (Figure 6). Furthermore, intra‐tumoural TLS tissues exhibited higher levels of CD4^+^Bcl6^+^ Tfh cells and CD4^+^Foxp3^+^ Tregs compared with adjacent tissues, indicating a robust and effective antitumour immune response (immune active) (Figure 7).

## 6 TEMPORAL HETEROGENEITY OF LIVER CANCERS

**FIGURE 8:**
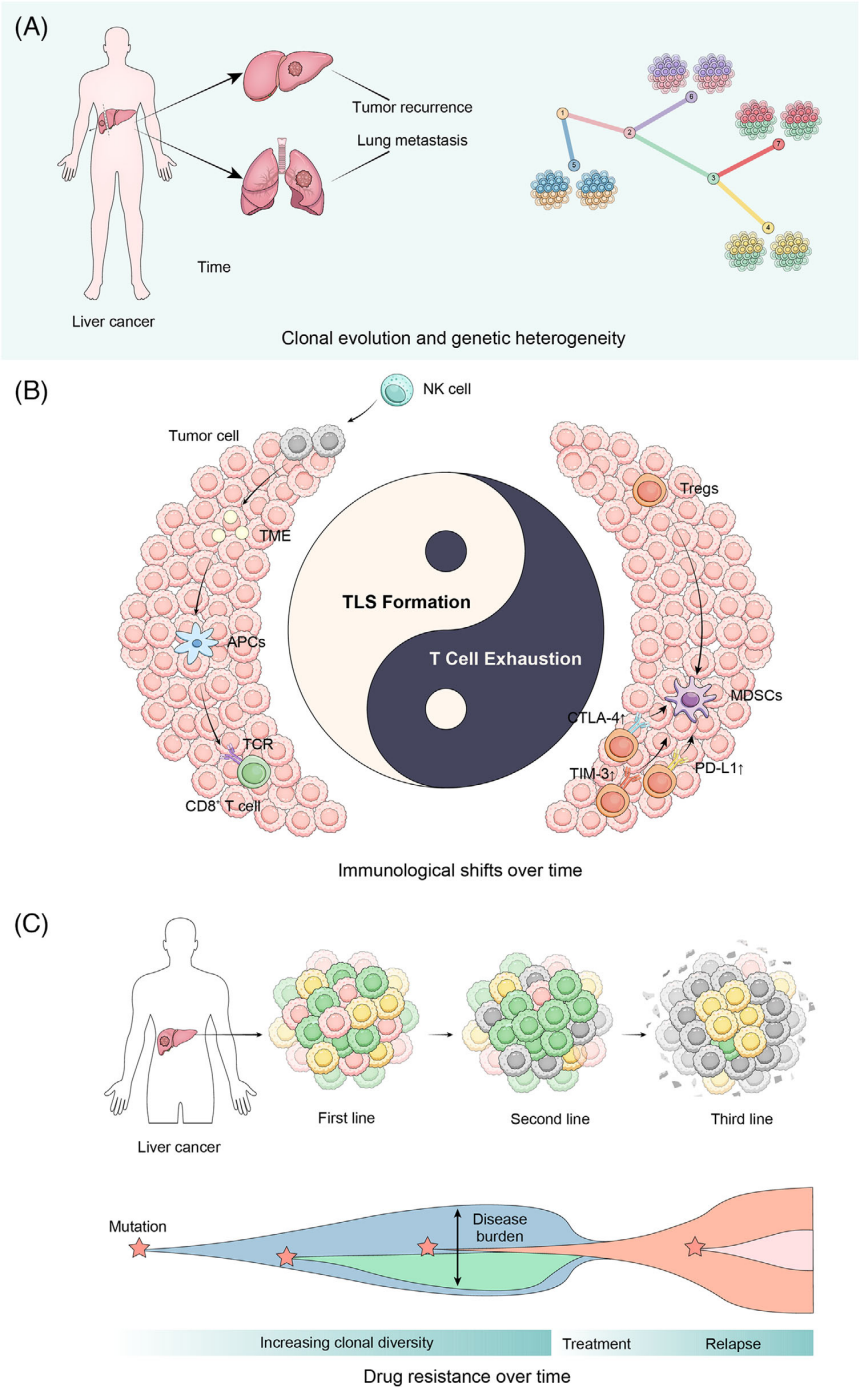
Temporal heterogeneity of liver cancers.

Temporal heterogeneity refers to the dynamic changes in tumour characteristics over time, influenced by genetic mutations, epigenetic modifications, metabolic shifts and interactions with the TME. In liver cancers, this temporal evolution significantly impacts tumour progression, treatment response and patient prognosis (Figure 8).

### 6.1 Clonal evolution and genetic heterogeneity in liver cancers

From an evolutionary perspective, clonal evolution tends to select for traits that enhance proliferation and survival, which may lead to invasion, metastasis and therapeutic resistance.^73^, ^74^ Whole‐genome sequencing analysis of 2658 tumour samples across 38 cancer types, including liver cancer, revealed that nearly all samples (95.1%) contained evidence of subclonal amplification. Within the same tumour, individual subclones frequently exhibit distinct mutational signature activities, and there are significant branching relationships among different subclones.^75^ Clonal evolution is a primary contributor to temporal heterogeneity in liver cancer, with distinct subclones emerging over time, particularly under therapeutic pressure. New mutations and selective expansion of particular subclones contribute to tumour recurrence and distant metastasis. Multi‐regional and longitudinal sampling techniques, such as spatial transcriptomics and single‐cell sequencing, have allowed researchers to track these clonal changes within primary and metastatic sites, shedding light on adaptive responses of tumour cells of liver cancers.^76^, ^77^

Tumour cells undergo continuous genetic alterations, leading to the emergence of diverse subclones with distinct mutations of liver cancers. Studies have shown that these subclones can evolve under therapeutic pressure, resulting in treatment resistance and disease recurrence. Recently, Yang et al.^78^ identified CD49f as a key marker for tumour‐initiating cells (TICs) in HCC, which recruit tumour‐promoting neutrophils via the CXCL2‒CXCR2 axis and create an immunosuppressive TME. Blocking CD155, a TIC‐specific vulnerability, enhances sensitivity to anti‐PD‐1 therapy, suggesting a potential therapeutic combination for HCC.

### 6.2 Immunological shifts over time in liver cancers

The cancer‐immunity cycle in liver cancers is a multi‐step process over time. In the early stages of liver cancer, the immune response tends to be more active. The rapid proliferation and high mutational burden of tumour cells activate innate immune cells like NK cells, which target and destroy tumour cells, releasing tumour‐associated antigens into the TME. These antigens are then captured by APCs, which migrate to secondary lymphoid organs to initiate adaptive immune responses. APCs present neoantigens to the T‐cell receptor (TCR) of CD8^+^ cytotoxic T lymphocytes via MHC class I molecules, leading to the activation of T cells that subsequently infiltrate the HCC tissue.^79^

However, as the tumour progresses, the immune environment becomes more suppressive, with a shift towards immune tolerance and evasion. This transition is facilitated by the recruitment of Tregs, MDSCs and the upregulation of immune checkpoint molecules, such as PD‐L1, CTLA‐4 and TIM‐3, which actively inhibit the immune response.^80^, ^81^

### 6.3 Drug resistance in targeted therapy and immune therapy of liver cancers

Targeted therapies are designed to specifically target tumour cells, avoiding the indiscriminate cell killing characteristic of traditional chemotherapy. Sorafenib, lenvatinib, regorafenib, tivantinib and cabozantinib are among the commonly used molecular targeted drugs in the treatment of liver cancer. Of these, sorafenib is currently an effective first‐line therapy for advanced HCC.^82^ In this part, we will use sorafenib resistance as a case study to explore the temporal heterogeneity of drug resistance in tumour‐targeted therapies.

Sorafenib is a multi‐target tyrosine kinase inhibitor (TKI) that exerts its antitumour effects through anti‐proliferation mechanisms by targeting key components of the Ras/Raf/MEK/ERK signalling pathway and blocks the activity of receptors such as the platelet‐derived growth factor receptor (PDGFR‐β), vascular endothelial growth factor receptor 2 (VEGFR‐2) and c‐KIT, thereby inhibiting tumour angiogenesis. However, despite its efficacy, only around 30% of HCC patients show a positive response to sorafenib, and those who do typically develop resistance within 6 months of treatment.^83^

In some patients, sorafenib treatment may initially show good efficacy, with tumour growth inhibited and quality of life improved. Due to the genetic heterogeneity of HCC, some tumour cells are inherently resistant to sorafenib with primary resistance, which can be partially attributed to the activation of EGFR downstream signalling.^84^, ^85^, ^86^ Recent studies have identified several mechanisms underlying acquired resistance to sorafenib, including crosstalk between the PI3K/Akt and JAK‒STAT pathways, activation of hypoxia‐inducible pathways and EMT.^87^, ^88^ Several studies also suggest epigenetic modifications, transport processes, regulated cell death and the TME in the initiation and development of sorafenib resistance in HCC.^89^, ^90^, ^91^, ^92^

Since the 2020 IMbrave150 study showed that the combination of atezolizumab and bevacizumab significantly improved OS over sorafenib in the first‐line treatment of unresectable HCC (19.2 months vs. 13.4 months, *p* < .001), the approach to systemic treatment for liver cancer has shifted from single‐agent TKI therapy to combination therapies with immune treatments.^93^ The HIMALAYA study first confirmed that dual immunotherapy with the PD‐L1 inhibitor durvalumab and the TIGIT inhibitor tiragolumab significantly improved prognosis in liver cancer patients compared to sorafenib monotherapy (16.4 months vs. 13.8 months, *p* < .001).^94^

One major cause of resistance to ICI therapy in liver cancer is primary resistance, where the tumour fails to respond to treatment from the outset. The ‘hot’ TME is characterised by high infiltration of cytotoxic T cells within the tumour and surrounding stroma, a high tumour mutational burden, elevated PD‐L1 and IFN‐γ expression, and is associated with a favourable response to ICI therapy.^95^ In contrast, a ‘cold’ TME is devoid of T‐cell infiltration and PD‐L1 expression. An excluded TME is characterised by the accumulation of cytotoxic T cells and other effector cells at the tumour margin, but these cells fail to infiltrate the tumour tissue, likely due to stromal barriers and aberrant vascular structures. Finally, an immunosuppressed TME exhibits moderate T‐cell infiltration, alongside immunosuppressive adaptations such as high expression of interleukin‐10 (IL‐10) and TGF‐β, as well as increased levels of Tregs, TAMs and MDSCs. Mutations in the Wnt/β‐catenin pathway, specific TP53 mutations, and loss of phosphatase and tensin homologue, leading to activation of the PI3K/AKT pathway, can further alter the TME and promote immune evasion in liver cancers, thereby contributing to primary resistance to immune therapy.^96^, ^97^, ^98^, ^99^ For instance, upregulation of β‐catenin enhances PD‐L1 expression, which subsequently reduces the cytotoxic activity of cytotoxic T cells, inhibits the recruitment of dendritic cell (DC) and T cells, and promotes immunosuppression through the expansion of Tregs.^100^ Furthermore, isocitrate dehydrogenase 1 (IDH1) mutations are frequently observed in cholangiocarcinoma and have been associated with immune evasion in mouse models.^101^

In contrast to tumour‐intrinsic mechanisms, extrinsic factors contribute to resistance to ICI therapy through specific cell clusters, cytokines, and metabolites derived from sources outside the tumour. Tregs employ several mechanisms to suppress immune responses, such as disrupting DC function or releasing immunosuppressive cytokines such as IL‐10, IL‐35 and TGF‐β, which in turn downregulate effector T‐cell activity.^102^ MDSCs, a heterogeneous population of pathologically activated immature cells, are key players in immunosuppressive networks. These cells effectively suppress T‐cell activity, thereby facilitating immune escape in malignant tumours.^103^ MDSCs can also impair the antigen‐presenting function of DC and promote the proliferation of Tregs, thereby enhancing tumour growth in HCC.^104^, ^105^ In addition to immunosuppressive cells and cytokines within the TME, alternative immune checkpoints such as TIM‐3 and LAG‐3 can contribute to resistance to ICI therapy. TIM‐3 has been found to be upregulated on TAMs in the TME following TGF‐β exposure, indicating an alternative mechanism of immune checkpoint inhibition that is not targeted by PD‐1/PD‐L1 or CTLA‐4 therapies. The coexpression of LAG‐3 and PD‐1 on tumour‐infiltrating lymphocytes creates a synergistic immunosuppressive effect, which may persist even after PD‐1/PD‐L1‐targeted ICI monotherapy, leading to treatment resistance.^106^, ^107^, ^108^, ^109^

## 7 CLINICAL APPLICATION AND CHALLENGES

The precise diagnosis and clinical management of liver cancer is based on and challenged by its spatial heterogeneities and temporal heterogeneities, which refer to the changing characteristics of the tumour across different regions and over time. Spatial heterogeneities and temporal heterogeneities in liver cancer significantly impact early diagnosis, treatment resistance, relapse and therapy responses, making personalised medicine strategies essential. Here, we discuss key areas where spatial and temporal heterogeneities could applied in the clinical practice and their potential challenges.

### 7.1 Knowledge about the cause and origin of liver cancers

**FIGURE 9:**
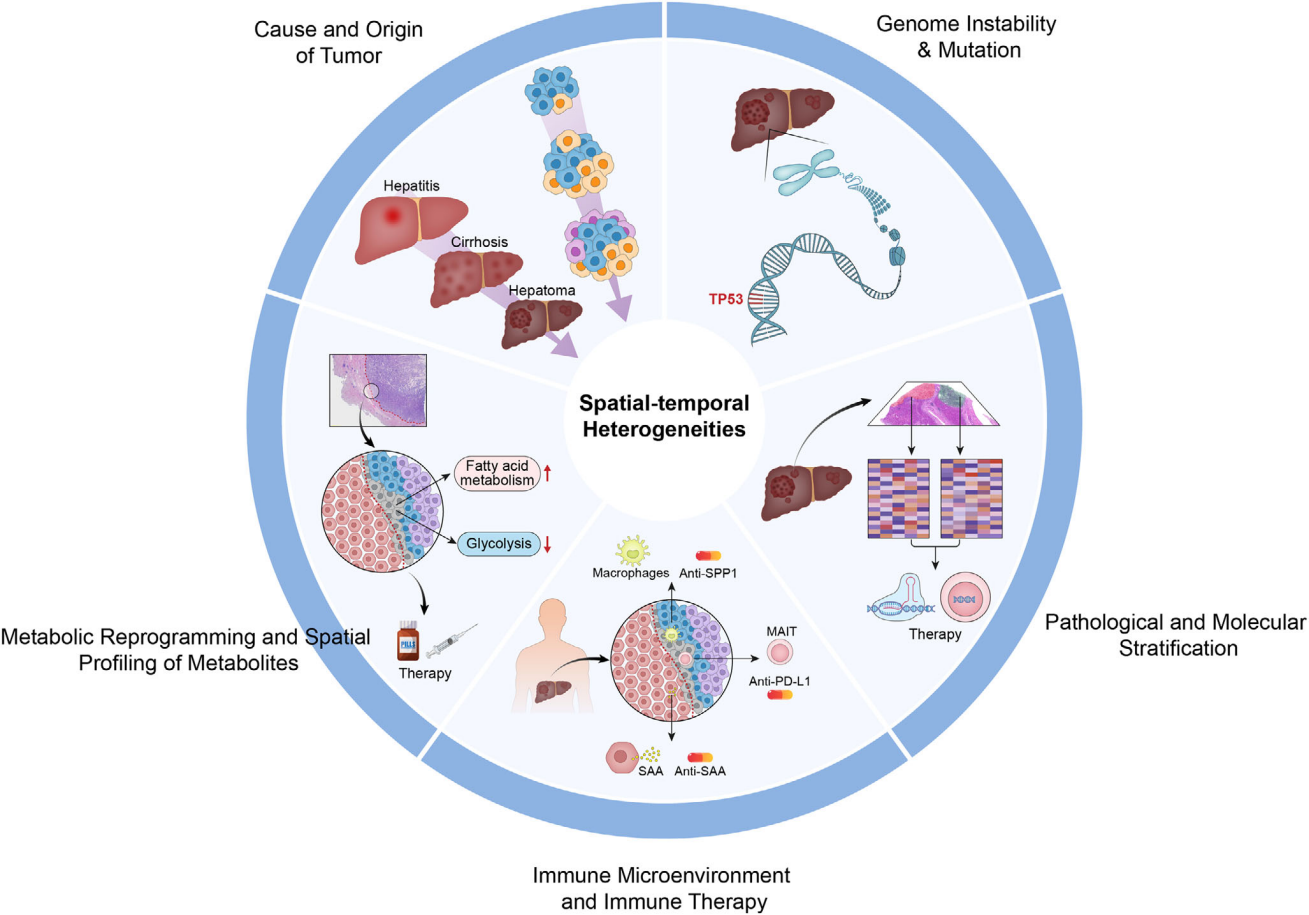
Role of spatial and temporal heterogeneities of liver cancer in the clinical application.

By integrating scRNA‐seq and spatial‒temporal omics technologies, we can achieve a comprehensive understanding of the multifactorial pathogenesis of liver cancers, leveraging single‐cell and spatially resolved data to capture gene expression, protein profiles and metabolites (Figure 9). These insights encompass a range of contributing factors, including chronic liver injury, liver fibrosis, cirrhosis, hepatitis virus infections (such as Hepatitis B and C), alcohol abuse, primary sclerosing cholangitis, and metabolic disorders such as metabolic dysfunction‐associated fatty liver disease.^110^, ^111^, ^112^, ^113^, ^114^, ^115^ By analysing tumour samples across different stages of disease, researchers can track how multifocal tumours evolve and how genetic and epigenetic changes accumulate over time. This temporal insight aids in distinguishing between primary liver tumours and distant metastases, providing valuable information for staging and treatment strategies.^6^, ^43^, ^116^ For example, Wu et al. conducted spatial transcriptomics analysis on tumour tissues from liver cancer patients using 10× Visium. They revealed the connection between TME remodelling and tumour metastasis through the distribution of PROM1^+^ and CD47^+^ cancer stem cells, providing new insights for potential tumour interventions.^117^

### 7.2 Defining genome instability and mutation

One of the key aspects that drives tumour progression, metastasis and therapy resistance is genomic instability, which manifests as mutations in key oncogenes and tumour suppressor genes. By integrating spatial transcriptomics and temporal analysis, researchers are now able to track how these genomic alterations evolve within the TME and across different stages of disease progression. This has profound implications for understanding the heterogeneity of liver cancers, identifying new biomarkers and developing more targeted treatments.

Core drivers (TP53, TERT and WNT signalling) and combinations of infrequent alterations in various cancer pathways cloud be captured in HCC and core driver genes (TP53, ARID1A, KRAS, SMAD4 and BAP1) with characteristic molecular alterations including fusion transcripts involving fibroblast growth factor receptor 2 (IGFR2) and the protein kinase A pathway, and IDH1/2 mutation could be found in ICC by multi‐omics tools such as whole exome sequencing.^118^ Temporal monitoring of driver mutations and drug resistance mutations is crucial for adapting treatment regimens and preventing treatment failure. Specifically, the acquisition of mutations in genes such as TERT, PIK3CA and ARID1A can be indicative of a poor response to standard chemotherapy. From a spatial perspective, unlike the variegated pattern observed in colorectal cancers, a significant proportion of HCC exhibits a clear isolation‐by‐distance pattern, where genetically similar regions are found in closer spatial proximity.^119^ Circulating tumour DNA (ctDNA), the feature of genome instability and mutation, are increasingly being used to track mutational changes and assess tumour progression in real time for HCC. This can allow for earlier intervention and more precise treatment planning. Dynamic trend of mutated ctDNA offers information reflecting HCC tumour biology including active states, stages, response to immune therapy and early recurrence.^120^

### 7.3 Pathological and molecular stratification of liver cancers

Spatial proteogenomics could provide pathological information about liver cancers, especially useful in precise identification of HCC, ICC or combined HCC and ICC. First‐line drugs for HCC include sorafenib, lenvatinib, atezolizumab plus bevacizumab, tremelimumab plus durvalumab and donafenib, whereas for ICC, the combination of gemcitabine and cisplatin is currently proposed as first line. By spatial transcriptomics, it is also potential to separate combined HCC and ICC cases into separate, combined and mixed subtypes with distinct pathological features.^121^, ^122^

Recently, our recent study established a heterogeneity landscape of HCC malignant cells by integrating scRNA and spatial transcriptomics datasets, and we identified three tumour cell subtypes: the ARG1^+^ metabolic subtype (metab‐subtype), the TOP2A^+^ proliferative subtype (prol‐phenotype) and the S100A6^+^ prometastatic subtype (EMT‐subtype).^123^ Further analysis showed that the S100A6^+^ subtype exhibits strong EMT features and metastatic potential. It forms a positive feedback loop with CAFs, promoting metastasis. Targeting this interaction with a CCN2 inhibitor offers an effective strategy for combating metastasis. Furthermore, the pathological and molecular stratification from spatial proteogenomic data could applied into the definition of liver cancers and clinical therapy. Sun et al. characterised multi‐regional and multi‐omics profiles (genomics, transcriptomics, scRNA‐seq, spatial transcriptomics and Immunohistochemistry, namely IHC) of paired primary and metastatic HCC samples from 182 patients.^43^ They found that tumour regions without Wnt‐related mutations were characterised by CAFs activation, T‐cell inflammation with terminal exhaustion, and an immunosuppressive TME, in contrast to Wnt‐mutated regions. These features were closely associated with immune therapy efficacy.

### 7.4 Spatial mapping of immune microenvironment and immune therapy

The TME in liver cancer is composed of immune cells, stromal cells and extracellular matrix components, all of which undergo spatial and temporal remodelling. Immune evasion is a hallmark of liver cancer, and understanding how immune cells evolve over time is key to improving immunotherapy outcomes. Spatial heterogeneity in immune cell infiltration, particularly TAMs, Tregs and MDSCs, plays a significant role in how tumours escape immune surveillance and therapy. Temporal changes in the immune microenvironment may impact response to immunotherapy, such as ICIs, which have shown promise in clinical trials.

By spatial transcriptomic mapping of the HCC microenvironment, Liu et al. identified a tumour immune barrier structure—a spatial niche where SPP1^+^ macrophages interact with CAFs to promote extracellular matrix remodelling.^124^ This structure is linked to the efficacy of immune checkpoint blockade. In preclinical models, blocking SPP1 or specifically deleting Spp1 in macrophages enhanced the effectiveness of anti‐PD‐1 treatment in mouse liver cancer, resulting in reduced CAFs infiltration and increased cytotoxic T‐cell infiltration. Additionally, the dysfunction and loss of cytotoxicity of MAIT cells enriched near the invasive zone was found to be closely linked to direct interactions with CD163^+^ PD‐L1^+^ TAMs and PD‐L1‐dependent mechanisms. This finding provides a basis for stratifying patients for aPD‐1/aPD‐L1 therapies targeting MAIT cells in HCC.^125^

Temporal changes in the immune checkpoint expression (e.g., PD‐L1 and CTLA‐4) in liver tumours are challenging to predict. Biopsy‐based analysis may fail to capture these dynamic changes, necessitating non‐invasive imaging and liquid biopsy approaches to track immune microenvironment shifts in real‐time. Su et al. reported that HCC patients receiving PD‐1 inhibitors combined with intensity‐modulated radiotherapy and antiangiogenic therapy showed better outcomes when baseline PD‐L1^+^ CTCs were <2, with higher objective response rate (56 .5% vs. 16.7%, *p* = .007) and longer OS (not reached vs. 10.8 months, *p* = .001).^126^ Additionally, a dynamic decrease in PD‐L1^+^ CTCs at 1 month post‐treatment was associated with a higher likelihood of achieving an objective response.

### 7.5 Tumour metabolic reprogramming and spatial profiling of metabolites

Tumour cells in liver cancer exhibit significant metabolic reprogramming, adapting to various conditions in the TME such as hypoxia, nutrient scarcity and therapeutic pressures. Temporal shifts and spatial heterogeneities in metabolic pathways, including glycolysis, oxidative phosphorylation and lipid metabolism, influence tumour growth, invasiveness and resistance to therapies, and could also be observed. Liver cancer cells exhibit metabolic plasticity, switching between different metabolic pathways to evade cell death and enhance survival under different TME stresses.

Spatial profiling of altered metabolites and lipids in HCC can deepen our understanding of tumour metabolic reprogramming and reveal potential metabolic vulnerabilities for therapeutic intervention.^127^, ^128^ By spatial metabolomics of HCC, arginine and its metabolites, spermine and spermidine, are significantly upregulated in tumour tissue, with their expression levels continuously increasing as the tumour grows. Additionally, choline and phosphatidylcholine metabolism undergo notable alterations during tumour progression.^128^ In the future, therapies targeting metabolic pathways identified through spatial metabolomics hold promising potential for clinical application.

The integration of spatiotemporal omics and digital pathology into clinical practice is a significant advancement in the personalised management of liver cancer. Spatiotemporal omics combines genomic, transcriptomic and proteomic analyses with spatial data to reveal the temporal evolution of tumour cells, immune cells and the extracellular matrix. Digital pathology, particularly when enhanced with machine learning and artificial intelligence‐based algorithms, allows for detailed analysis of tissue slides, capturing both spatial distribution and temporal changes in tumour characteristics.^129^ Shi et al. analysed whole‐slide images of HCC patients and developed a ‘tumour risk score’ (TRS) to predict patient outcomes.^130^ They integrated TCGA multi‐omics data to explore the underlying mechanisms of TRS. Key features of TRS included sinusoidal capillarisation, prominent nucleoli and karyotheca, nucleus/cytoplasm ratio and inflammatory cell infiltration. The TCGA data suggested that TRS is linked to tumour immune infiltration and genetic mutations, such as FAT3 and RYR2. Spatial transcriptomics and scRNA‐seq have allowed for the detailed mapping of liver cancer tumours over time.

These technologies can identify subclonal populations within a tumour and map their temporal evolution, improving diagnostic accuracy and informing treatment strategies. For example, real‐time monitoring of tumour dynamics using digital pathology can help identify the emergence of drug‐resistant clones, enabling timely adjustments to treatment plans.

## 8 CONCLUSION AND FUTURE PERSPECTIVE

In summary, although pathological observations have extensively documented the spatial heterogeneity of solid tumours, the significance of these differences remains unclear. Recently developed high‐throughput sequencing technologies, such as spatial transcriptomics and spatial proteomics, offer promising capabilities for capturing the spatial distribution of cell subpopulations and local networks of intercellular communication in situ. Spatial heterogeneity in liver cancers is reflected in eight key macroscopic features: tumour size, number, segmental distribution, gross marginal morphology, PVTT, CTC, LN metastasis and distant metastasis. It is also evident in four major pathological features: tumour budding, MVI, tumour capsule and margin area, each exhibiting distinct genomic and transcriptional characteristics. Distinct heterogeneities were also observed in the TME, including mixed and interplaying antitumour and protumour components, an ‘immunologically hot’ or ‘cold’ tumour phenotype, an immunosuppressive microenvironment around the borderline, and the presence and maturity of TLS. Furthermore, future studies should also define the roles of the adjacent liver tissues (often damaged in HCC patients due to cirrhosis or steatosis) in tumour heterogeneity and treatment resistance.

Our initial studies systematically analysed the spatial heterogeneities of liver cancer and identified a 500 µm‐wide zone centred around the tumour boundary of human liver cancers, referred to as the ‘invasive zone’. This zone is characterised by strong immunosuppression, metabolic reprogramming and severely damaged hepatocytes detected. The ecosystem comprising tumour cells, hepatocytes and macrophages was identified in detail. Identifying the invasive zone provides new insights into the spatial heterogeneity of liver cancer and offers valuable biological insights for developing novel and more effective therapeutic approaches for solid tumours. Although Stereo‐seq offers high nanoscale resolution (220 nm/spot) to capture transcripts from a few hundred spots per cell, it remains challenging to accurately identify cell boundaries and capture precise transcript information at the single‐cell level. In our study, the raw spatial expression matrix, obtained at nanoscale resolution, was converted into cell‐sized pseudo‐spots (25 µm^2^), consisting of 50 × 50 bins per pseudo‐spot. These pseudo‐spots were then assigned to specific cell types based on the highest probability. Despite these efforts, spatially resolving single‐cell genetic information and precisely inferring cell types remains difficult due to current technical limitations. However, future technological advancements may address these challenges.

Emerging sequencing technologies such as DBiT‐seq, spatial‐CITE‐seq, spatial ATAC‐RNA‐seq, CUT&Tag‐RNA‐seq, SPOTS, SM‐Omics, Stereo‐CITE‐seq and spatial RNA‐TCR‐seq offer new opportunities and perspectives for investigating the spatial and temporal heterogeneities of liver cancers.^131^, ^132^, ^133^, ^134^, ^135^, ^136^, ^137^ Formalin‐fixed paraffin‐embedded (FFPE) spatial transcriptomics serves as a valuable tool for studying liver cancer, preserving tissue architecture and enabling the analysis of gene expression within the context of the TME.^137^ It enables the mapping of immune cell infiltration and gene expression variability across different tumour regions and stages, such as comparing the tumour core versus invasive margins or evaluating changes over time, such as 5 years before versus 5 years after treatment. In liver cancer research, FFPE spatial transcriptomics is an ideal tool for comparing spatial and temporal heterogeneities in tumour recurrence, metastasis and immune therapy response. In addition to the core functions of spatial transcriptomics, Stereo‐CITE‐seq can capture the expression of over a hundred markers in situ, combining both transcriptomic and proteomic data on the same chip.^138^ Recently, Qiu et al. draws on mathematical models from multiple interdisciplinary fields, including physics, geography and economics, and has pioneered the development of the three‐dimensional spatiotemporal modelling toolkit, Spateo.^139^ This toolkit enables the precise reconstruction of organ three‐dimensional structures and the systematic quantification of spatiotemporal dynamics with potential application in the discovery of spatial heterogeneity and cellular communication in situ of solid tumours.

In summary, although significant advances have been made in understanding the spatial heterogeneity of liver cancers, key aspects of its biological relevance remain unclear. The integration of high‐throughput sequencing technologies, such as spatial transcriptomics and proteomics, provides powerful tools to capture the intricate spatial distribution of tumour subpopulations and their local microenvironments. Notable macroscopic features of liver cancer heterogeneity include tumour size, segmental distribution and metastasis, while key pathological features such as tumour budding, MVI and immune cell infiltration reflect distinct genomic and transcriptional profiles.

Our findings on the ‘invasive zone’ in liver cancer offer crucial insights into the spatial heterogeneity, revealing a region with significant immunosuppression and metabolic reprogramming. This zone, identified through detailed spatial analysis, provides a valuable framework for understanding the TME and the complex interplay between tumour cells, hepatocytes and immune cells. However, the current technical limitations, such as the difficulty in precisely mapping single‐cell transcriptomics, highlight the need for further advancements in spatial resolution and cell‐type identification.

Emerging technologies such as DBiT‐seq, spatial‐CITE‐seq and Stereo‐CITE‐seq hold great promise in refining our understanding of liver cancer heterogeneity by offering multi‐layered insights into gene expression, immune infiltration and cellular communication. FFPE spatial transcriptomics further enhances our ability to study the TME in preserved tissue samples, allowing for longitudinal studies on tumour progression, recurrence and treatment responses.

Looking ahead, the continued development of advanced spatiotemporal modelling tools, such as the Stereo‐CITE, will be instrumental in reconstructing organ‐level three‐dimensional structures and quantifying dynamic tumour evolution. These innovations will not only elucidate the complexities of liver cancer heterogeneity but also pave the way for the development of more personalised and effective therapeutic strategies. As spatial and temporal omics technologies continue to evolve, they hold the potential to revolutionise our approach to understanding liver cancer and other solid tumours, transforming both diagnostic and therapeutic paradigms.

## AUTHOR CONTRIBUTIONS

Conceptualization, Jian Zhou, Xinrong Yang, Liang Wu, and Dan G Duda; Investigation and Writing Original Draft, Jiayan Yan, Zhifeng Jiang, Shiyu Zhang, Qichao Yu, Yijun Lu, and Runze Miao; Investigation, Jiayan Yan, Zhifeng Jiang; Writing ‐ Review & Editing, Dan G Duda, Jian Zhou; Visualization, Shiyu Zhang, Runze Miao; Funding Acquisition, Jian Zhou, Xinrong Yang, and Jiayan Yan; Supervision, Jian Zhou, Zhaoyou Tang, Jia Fan, Xinrong Yang, Liang Wu.

## CONFLICT OF INTEREST STATEMENT

The authors declare they have no conflicts of interest.

## ETHICS STATEMENT

All patients provided informed consent for the collection of clinical information, and the tissue collection protocols were approved by the Institutional Review Board [approval B2018‐018(3)] at Zhongshan Hospital, Fudan University.
